# A novel zinc complex with antibacterial and antioxidant activity

**DOI:** 10.1186/s13065-021-00745-2

**Published:** 2021-03-15

**Authors:** Yun Zhang, Xiaojing Li, Jia Li, Md. Zaved Hossain Khan, Fanyi Ma, Xiuhua Liu

**Affiliations:** 1 Key Laboratory of Natural Medicine and Immuno-Engineering of Henan Province, School of Pharmacy, Henan International Joint Laboratory of Medicinal Plants Utilization, College of Chemistry and Chemical Engineering, Henan University, Kaifeng, 475004 China; 2 Department of Chemical Engineering, Jashore University of Science and Technology, Jahsore, 7408 Bangladesh

**Keywords:** Zinc-glucose-citrate complex, Synthesis, Characterization, Fluorescence, Antibacterial activity

## Abstract

**Background:**

In order to enhance the antibacterial activity and reduce the toxicity of Zn^2+^, novel complexes of Zn(II) were synthesized.

**Results:**

A water-soluble zinc-glucose-citrate complex (ZnGC) with antibacterial activity was synthesized at pH 6.5. The structure, morphology, characterization, acute toxicity, antibacterial and antioxidant activities, and in situ intestinal absorption were investigated. The results showed that zinc ion was linked with citrate by coordinate bond while the glucose was linked with it through intermolecular hydrogen bonding. The higher the molecular weight of sugar is, the more favorable it is to inhibit the formation of zinc citrate precipitation. Compared with ZnCl_2_, ZnGC complex presented better antibacterial activity against *Staphylococcus aureus* (*S. aureus*, Gram-positive) and *Escherichia coli* (*E. coli*, Gram-negative).

**Conclusions:**

The results of acute toxicity showed no obvious toxicity in this test and in situ intestinal absorption study, suggesting that ZnGC complex could be used as a potential zinc supplement for zinc deficiency.

## Introduction

Currently, numbers of serious problems in clinics were caused by various bacterial infections. The multi-drug resistance limits the progress of conventional antimicrobial agents in the clinics. More and more antibiotics have been explored in order to overcome these issues [1]. Some heavy metal ions with biocidal action such as silver, zinc or copper, have been used as inorganic metal antibacterial materials. As most of the substances are toxic, zinc is one of essential dietary trace metals required for a number of physiological and biochemical functions in human body. Zinc deficiency can lead to series of hazardous influence to immune systems [2, 3]. Furthermore, zinc itself exhibits antibacterial efficacy. Zinc chloride can effectively inhibit the growth of almost all strains causative of halitosis and periodontal disease resulting in a direct decrease of the bacterial production [4, 5]. Zinc ion possibly inhibits the growth of plaque formation and exhibits a promising potential to be used as an antibacterial agent in future dentifrices and mouth rinses [6]. Zinc containing wound dressings are commonly used for topical applications which enhance healing of chronic and acute wounds [7–9]. Meanwhile, there are also many evidences that zinc can provide anti-infective action in the damaged skin or tissues with little side effects [10–15]. Free zinc ion accelerates hospital acquired infections by increasing the virulence of *Streptococcus pyogenes* and inducing intercellular adhesion of *Staphylococcus epidermidis* and *Staphylococcus aureus* [16]. Therefore, the development of biofilms chelated zinc represents a potential therapeutic approach for combating biofilm growth in a wide range of biofilm-related infections. Additionally, zinc ion has protein-precipitating action resulting in tissue contraction, corrosion and a chemical fixative effect. High concentrations of zinc chloride ingestion cause obstructive scarring in the pylorus and mild corrosion of oropharynx or oesophagus. Zinc chloride has an inherent degree of toxicity with a great ability to permeate tissues. Ingestion of zinc chloride might cause the stimulatory action on the mucous membranes of gastrointestinal tract with vomiting, abdominal pain, and diarrhea. In order to reduce the toxicity and irritation of free zinc ion, various zinc complexes come to wide attention [17–19].

Trisodium citrate is a multipurpose class of ligand having notable biological and chemical activities. Therefore, the design and preparation of new complexes with zinc and trisodium citrate, which aims to improve their proprieties through the discovery of new structures, is still a great scientific challenge. Further continuing research on our previous work [20–22], a novel zinc complex (ZnGC) was synthesized in this study by using Zn^2+^, glucose and trisodium citrate. The structural, chemical components, characterization, acute toxicity and in situ intestinal absorption study of the complex was performed and evaluated. In order to investigate whether the complex formed, it was heated at 130 °C for 1 h, which resulted a fluorescence phenomenon observed under 365 nm ultraviolet light. The antibacterial activity was investigated and confirmed against both Gram-positive *Staphylococcus aureus* (*S. aureus*) and Gram-negative *Escherichia coli* (*E. coli*) strains [23]. The ZnGC complex has a potential to be used as a candidate for zinc supplements and the application in antibiosis material in biomedical fields.

## Material and methods

### Materials and animals

All chemicals were purchased from Sigma-Aldrich Co., Ltd. or Tianjin Kemiou Chemical Reagent Co., Ltd., and all chemicals used were of the analytical reagent grade (AR). Deionized water (pH 7.0, 18.2 mΩ) was used for all experimental works.

Male and female Kunming mice (20–24 g), male Wistar rats (250–300 g) and the normal diet were purchased from Center of Laboratory Animals of Henan Province (Zhengzhou, China). All animals were housed with maintained temperature at 22 ± 3 °C under natural light/dark conditions and free access to food and water. All animal experimental procedures were approved by Henan University Institutional Animal Care and Use Committee.

### Synthesis of ZnGC

The mixture of 8.0 mmol glucose and 2.0 mmol trisodium citrate dihydrate was blended in 40 mL deionized water for 5 min. Then, 2.2 mmol ZnCl_2_ was subsequently dropped into the solution, and 1 mol L^−1^ NaOH or HCl was used to adjust pH to 6.5 with constant stirring at 50 °C for 24 h. The white precipitate was observed when ethanol was added until its concentration reached approximately 80% vol. The prepared solution was then centrifuged at 4300 × *g* for 2 min, the obtained sediment was washed with 80% ethanol three times, and then it was dissolved in water and lyophilized (Christ Delta 1–24 LSC plus, German). This zinc complex obtained was named ZnGC.

### Determination of zinc, glucose and citric acid contents

#### Zinc content

The quantification of zinc content was detected by using ICP-AES system (Optima 2100DV, Perkin Elmer, USA).

#### Glucose content

The glucose content of ZnGC complex was detected following Galant with minor modification [24]. The samples were dissolved in 2 mL 1 mol L^−1^ HCl, then 50 mL water and 5 mL 0.05 mol L^−1^ iodine standard solution were added in. 1 mol L^−1^ NaOH solution was slowly dropped into the solution until the color became to yellowish. After being reacted in the dark for 15 min, titrate to the end point immediately by Na_2_S_2_O_3_ standard solution with adding 2 mL 1:1 HCl. The glucose content (w/w) in the samples were calculated according to the following equation.

1 $${\text{glucose\% }} = \frac{{(Mol_{{I_{2} }} - 2 \times Mol_{{Na_{2} S_{2} O_{3} }} ) \times M_{glu\cos e} }}{sample\,weight} \times 100\%$$

Note: M_glucose_ = 180.157.

#### Citric acid content

Citric acid was determined by the high-performance liquid chromatography (HPLC) method [25], which performed on an Agilent 1260 system (Agilent, USA) with a diode array detector (DAD) and the ChemStation (Agilent Technologies, USA) software for data processing. The chromatographic separation was carried out on a reversed-phase column packed with octadecylsilane phase particles (250 × 4.6 mm, 5 μm, Agilent Eclipse XDB-C18) at 25 °C. The mobile phase consisted of acetonitrile and 0.5% ammonium dihydrogen phosphate (phosphoric acid regulates pH = 3) at 5:95 (v/v). The flow rate was set at 1.0 mL min^−1^, the detection wavelength at 210 nm, and the sample injection volume was 10 μL.

For the stock solution of the citric acid standard was dissolved in water at a concentration of 3.0 g L^−1^. The samples were dissolved in 1 M HCl at a concentration of 4.0 g L^−1^ by appropriate dilutions. All samples and standards were injected three times each and mean values were used [26].

### Stability of ZnGC complex

ZnGC complex dissolved in ultra-pure water was dialyzed using 1000 Da dialysis tube in a beaker containing 200 mL ultra-pure water with stirring. 1 mL of dialysate was taken at 1, 12, 24 and 48 h respectively, and 1 mL of ultra-pure water was added after each withdrawal. Then, the content of zinc ions in the dialysate were determined by ICP-AES system.

### Physicochemical characterization

#### Scanning electron microscopy (SEM) and energy dispersive spectrometer (EDS)

SEM and EDS (JSM-7001F JEOL, Japan) were used to inspect the morphology of ZnGC, analyzed the element distribution to determine whether the energy was converted, transformed or transported.

#### Transmission electron microscopy (TEM)

Transmission electron microscopy (TEM, JEM2100Plus, JEOL, Japan) were used to inspect the surface morphology of ZnGC.

#### Fourier transform infrared (FT-IR) spectroscopy

The FT-IR spectra were recorded in the range of 4000–400 cm^−1^ wavelength with KBr discs using infrared spectrometer (Thermo Scientific Nicolet iS 50, USA) and the structural characteristics of glucose, trisodium citrate dihydrate and ZnGC complex were investigated.

#### X-ray diffraction (XRD)

An X-ray powder diffractometer (Bruker D8 Advance, Germany) was used to characterize with Cu Kα radiation.

#### X-ray photoelectron spectroscopy (XPS)

The analysis of energy-disperse X-ray was performed on energy-disperse X-ray instrument (Thermo esca lab 250XI, Thermo Scientific Co. Ltd., USA).

#### Thermal gravimetric analysis (TGA)

Thermal studies (TGA/DTG) were carried out on TGA/DSC 3 + Thermogravimetric Analyzer (Mettler Toledo Corp., Zurich, Switzerland) and the samples were heated up to 1000 °C at rate of 10 °C·min^−1^ in an inert nitrogen atmosphere.

#### NMR

^1^H and ^13^C spectra were recorded in D_2_O as solvent on a Bruker Avance Digital 300 MHz NMR spectrometer (Switzerland).

#### Fluorescence spectroscopy

In order to prove the conjugation of zinc with glucose and trisodium citrate, ZnGC complex, both the complex and mixture of ZnCl_2_, glucose and trisodium citrate of same mass were heated directly in muffle (Barnstead 2555, U.S.A) at 130 °C respectively and kept for 1 h. Then, fluorescence was observed under 365 nm ultraviolet light.

### Antioxidant activity

The scavenging activity of hydroxyl free radical of ZnGC complex was determined [27]. ZnGC complex sample with different concentrations (0.5, 1.0, 1.5, 2.0, 2.5, 3.0 mg/mL) was mixed to a 1.0 mL H_2_O_2_ solution (9 mM) and 1.0 mL FeSO_4_ (9 mM) solution and incubated at 37 °C for 10 min. Then, 1.0 mL salicylic acid–ethanol solution (9 mM) was added and reacted at 37 °C for 30 min. Distilled water replaced salicylic acid–ethanol for the control, and distilled water replaced the sample solution for the blank. The hydroxyl radical was detected by monitoring absorbance at 517 nm, and the hydroxyl radical scavenging activity (HRS%) was calculated as:

2 $${\text{HRS\% }} = \left[ {1 - \frac{{{\text{A}}_{{{\text{sample}}}} - {\text{A}}_{{{\text{control}}}} }}{{{\text{A}}_{{{\text{blank}}}} }}} \right] \times 100\%$$

where A_sample_ is the absorbance of the sample group, A_control_ is the absorbance of the control, and A_blank_ is the absorbance of the blank sample.

### Antibacterial study

Antibacterial activity of ZnGC complex was tested against *S. aureus* (Gram-positive bacteria) and *E. coli* (Gram-negative bacteria) using agar well diffusion method [28]. Microbial strains of *Escherichia coli* (*E. coli*, ATCC8899) and *Staphylococcus aureus* (*S. aureus*, ATCC25923) were obtained from School of Life Sciences, Henan University. Briefly, the required nutrient agar medium was prepared by mixing peptone (5.0 g), beef extract (3.0 g), sodium chloride (5.0 g) and agar (15.0 g) in 1000 mL distilled water, and the pH of the medium was adjusted to 7.0. *E. coli* and *S. aureus* were cultured in 125 mL of Luria–Bertani (LB) broth at 37 °C for 20 h, and the bacterial cells were centrifuged for 10 min at 3500 r/min. The suspension of bacteria cell had a final density of approximately 4 × 10^8^ colony forming unit (CFU) per mL. Metal borers were used to produce wells in the agar plates. Different concentrations in the range of 7.0–18.0 mmol L^−1^ of ZnGC complex were placed in these wells and incubated at 37 °C for 24 h. The same concentration of ZnCl_2_ was used as positive control. The diameters of inhibition zones were measured using a Vernier caliper. The experiments were repeated three times for each bacterial strain and average values of zone of inhibition were recorded.

### Minimum inhibitory concentration (MIC)

MIC is an efficient method to determine antibacterial activity of any material, quantitatively. To determine MIC of ZnGC complex, serial dilution method was employed. 1 mL ZnGC complex was taken in sterilized test tubes containing 1 mL of bacterial (*S. aureus* or *E. coli*) solutions with a turbidity of 0.5 McFarland turbidity standard. The test tubes only with bacterial culture were taken as control. All test tubes were then kept in an incubator at 37 °C for 24 h [29].

### Acute toxicity study

Acute toxicity of ZnGC complex was tested by oral administration which was performed according to the preliminary experiment [30]. For each administration, mice were divided into five dose-groups and one control group (10 mice, half males, and half females for each group). The mice were administered with increasing doses at 500, 700, 900, 1100, and 1300 mg kg^−1^ body weight (BW). The control group was provided with the isotonic saline solution by oral administration. All mice were permitted for food and water freely and were observed for 14 days after administration of doses.

### In situ intestinal absorption study

Male Wistar rats weighing 250–300 g were supplied by Center of Laboratory Animals of Henan Province (Zhengzhou, China). In situ intestinal absorption property of ZnGC was investigated by means of single-pass intestinal perfusion technique as previously described [31, 32]. All rats were fasted 16 h and anesthetized with sodium pentobarbital (40 mg kg^−1^ body weight). About 3 cm midline abdominal incision was made and an intestinal segment (duodenum to ileum) was cannulated at both ends. The tubes were connected to an infusion pump and the chosen intestinal segment. After rinsed with saline at 37 °C to remove the chyme and other food particles, the surgical area was covered with pledget to prevent peritoneal liquid evaporation and heat loss. Then, the experiment was started with preliminary perfusion of ZnGC samples dissolved in saline at the flow rate of 4 mL min^−1^. The irrigation was balanced for 10 min and then the flow velocity was reduced to 1 mL min^−1^. At this point, it was denoted as 0 with recording volume of the solution (V_0_). 1 mL outlet perfused samples were collected at 0.25, 0.5, 1.0, 1.5, 2, 3 h respectively and 1 mL saline was added after each withdrawal. The collected samples were detected by ICP-AES systems for zinc content immediately. After termination of the experiment, the perfused intestinal segment was measured without stretching. For each group, the initial volume (V_0_) and the endpoint volume (V_t_) was determined on each animal used. Drug solutions were pre-warmed at 37 °C before administration into the intestinal lumen. The body temperature was maintained during anesthesia by heating with a lamp. Rats were sacrificed humanely at the end of experimentation. ZnCl_2_ was taken as the reference. The absorption rate constant Ka and absorption percentage P were calculated according to Eqs. (3) and (4).

3 $$ \ln X = \ln X_{0} - K_{a} {\text{t}} $$

4 $$ P\left( {\% \cdot h^{ - 1} \cdot cm^{ - 2} } \right) = \frac{{C_{0} \times V_{0} - C_{t} \times V_{t} }}{{C_{0} \times V_{0} \times t \times L \times W}} \times 100\% $$

where: X_0_ was the initial dosage in the perfusion, X_t_ was the dosage at time t; C_0_ was the initial drug concentration in the perfusion, and C_t_ was the drug concentration at time t; V_0_ was the initial volume of perfusion, V_t_ was the volume of perfusion liquid at time t. L was the length of intestinal segment at both ends of intubation, while W was the width of the intestine segment after being opened.

### Statistical analysis

Each sample was repeated at least triplicates, and the resulting data were analyzed by one-way analysis of variance (ANOVA), and presented as mean values ± standard deviations (SD).

## Results and discussion

### Contents of zinc, glucose and citric acid

To find the optimal condition for synthesizing ZnGC complex, the effects of pH, temperature and reaction time were investigated (data not shown). On the basis of the NMR and HPLC results, the optimal condition was casting the complex at pH 6.5, 50 °C for 24 h. The obtained ZnGC complex was white in powder which was easily dissolved in water. The contents (w/w) of zinc, glucose and citric acid were approximately (18.02 ± 0.54)%, (6.60 ± 0.06)% and (48.05 ± 0.07)% respectively. The results indicated that the sample may also contain Na^+^ and crystal water. The mole ratio of Zn: glucose: citric acid was approximately 7.5: 1.0: 6.8 in ZnGC complex, suggesting that macromolecular polymers were formed.

### Stability of ZnGC complex

The content of zinc in dialysate detected at 1, 12, 24, 48 h were 0, 0.615, 1.253 and 4.392 mg L^−1^, respectively. After dialyzing at 4 °C for 48 h, approximately 3.0% zinc was penetrated through membrane, indicating that ZnGC complex formed a relatively stable polymer [33]. Zinc and hydrogen ions could slowly exchange in an aqueous solution, resulted in dissociation of Zn^2+^. It was speculated that hydroxyl groups in glucose participate in the coordination hindered the binding of zinc ions to citric acid to form insoluble zinc citrate. According to our previous study [20], the longer the chain of the polysaccharide involved in the coordination, the more difficult the zinc citrate precipitation formed.

### Characterization of ZnGC complex

#### SEM and TEM

**Fig. 1:**
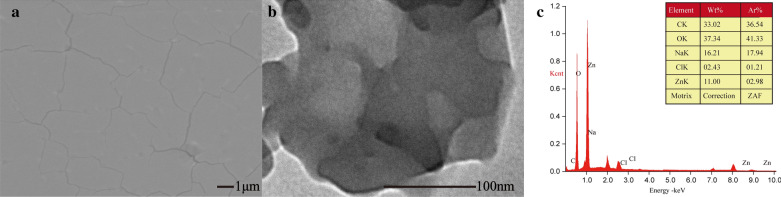
Morphology by SEM (**a**) and TEM (**b**), and energy spectrum (**c**) of ZnGC

Figure 1a and b show the surface morphologies of ZnGC complex by SEM and TEM respectively. Figure 1a exhibits a smooth surface, homogeneity and compact thick flake-like morphology. The TEM image (Fig. 1b) also showed the structural conformance and homogeneous in composition. The results revealed the existence form of zinc in ZnGC complex stated in ionic condition and no nanoparticles formed. Energy dispersive spectrometer (EDS) analysis also indicated that there was a significant zinc signal in ZnGC complex (Fig. 1c).

#### FT-IR

**Fig. 2:**
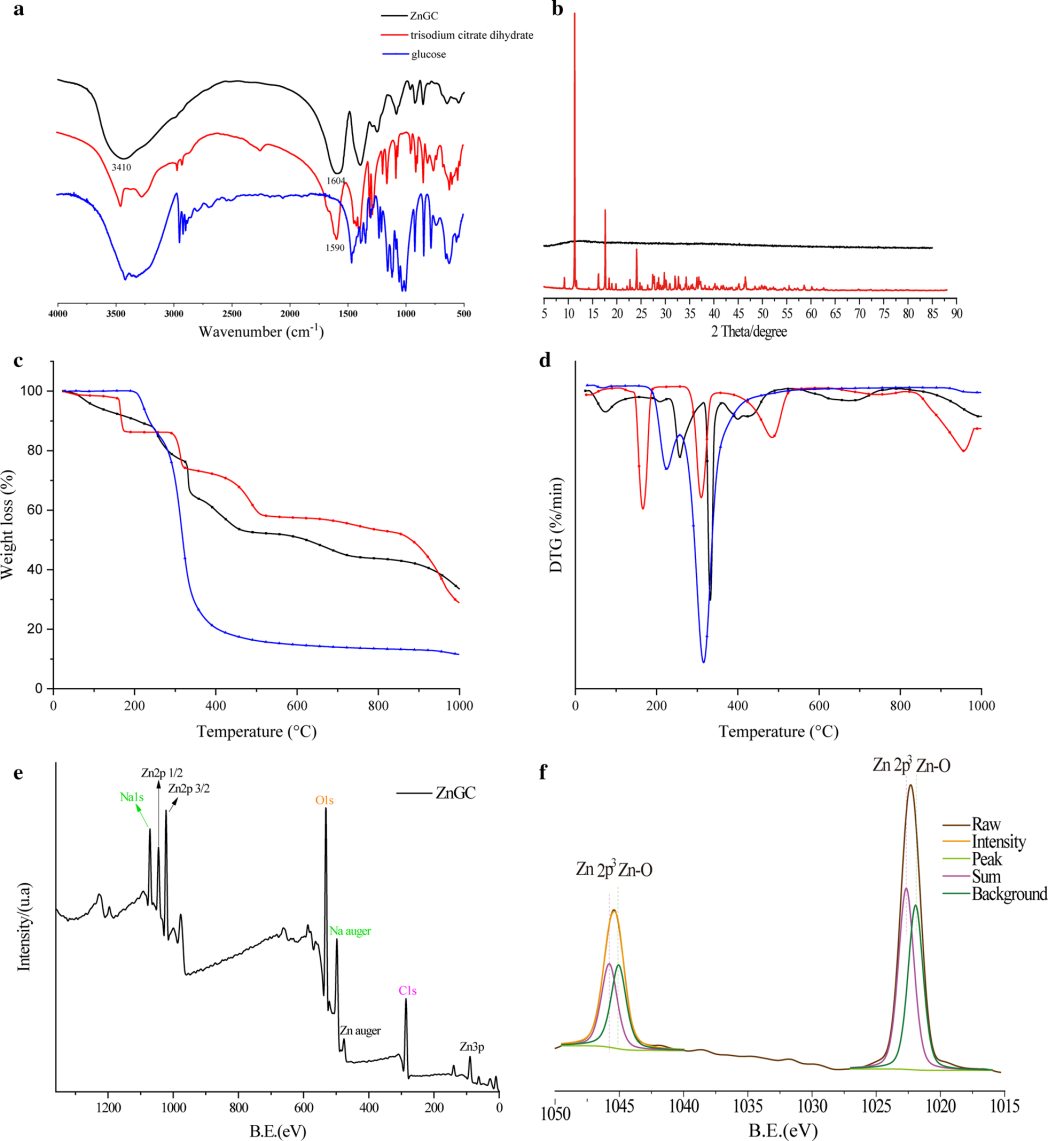
FT-IR spectra (**a**); XRD (**b**); thermal gravimetric analysis including TGA (**c**) and DTG (**d**); XPS spectra for ZnGC (**e**) and zinc 2p (**f**)

Figure 2a shows the FT-IR spectra of ZnGC, glucose and trisodium citrate dihydrate. Generally, a broad peak observed in ZnGC approximately 3410 cm^−1^ corresponded to O–H stretching, which indicated that intermolecular hydrogen bonding presented in the complex. The C–H stretching vibrations of glucose and trisodium citrate occurred in the range of 2960–2800 cm^−1^, yet disappeared in ZnGC complex. The results suggested the chelation of trisodium citrate ligand occurs via oxygen atoms of carbonyl groups and the bindings of zinc lowered the strength of –CH. The carboxylate group was able to coordinate to metal ions by different modes. When the difference in wavenumbers between the asymmetric and symmetric stretching vibrations of carboxylate groups, (Δν = ν^as^COO^−^—ν^s^COO^−^), was larger than 200 cm^−1^, I-monodentate chelate was formed. II-bidentate coordination occurred, when Δν was considerably smaller than 200 cm^−1^ [34]. ZnGC exhibited two absorption bands at 1604.72 and 1394.52 cm^−1^ for the ν^as^COO^−^ and ν^s^COO^−^ (Δν = 210.20 cm^−1^) respectively, which suggests that the carboxylate groups act as monodentate chelate. The new stretching frequency at 915.0 cm^−1^ was attributed to the bending vibrations of the hydroxyl groups in Zn–OH [35]. Hence, the FT-IR spectrum confirmed that the zinc ions were bonded with glucose and trisodium citrate to form a novel ZnGC complex.

#### X-ray diffraction (XRD)

X-ray diffraction pattern of ZnGC illustrates in Fig. 2b. ZnGC complex showed no sharp peak suggesting that the nature of ZnGC was amorphous and no nanoparticles was formed. These results indicated that intermolecular citrate zinc cross-linking increased and a conformational changed from crystalline order to disorder while zinc was coordinated to citrate. The spatial volume of sodium citrate increased as loading zinc and glucose, and the average particle size was accordingly increased. This could be due to complexation of zinc ion with trisodium citrate and glucose by coordination bond, which was in accordance with the results of TEM and FT-IR [36].

#### Thermogravimetric analysis

The TGA and DTG curves of ZnGC, glucose and trisodium citrate dihydrate are shown in Fig. 2c and d respectively. Trisodium citrate dihydrate showed the weight loss in four steps. Firstly, approximately 15% weight loss was observed at approximately 170 °C, which could be due to the loss of absorbed moisture. In the second and third stage, the major thermal degradation (approximately 10 and 15% weight loss) was observed at approximately 310 and 485 °C respectively, which might be caused by the breakdown of the chemical structure. The final event of trisodium citrate dihydrate occurred at 956 °C indicating that the sample was completely ashed. The total weight loss of trisodium citrate dihydrate was approximately 70.70%. The glucose sample showed no moisture loss and decomposition was delayed by approximately 315 °C, which illustrated that the glucose maintained thermal stability after 360 °C. The total weight loss of glucose was 88.41%.

ZnGC complex showed intricate thermal properties. The first endothermic depression lost approximately 10% weight at approximately 75 °C, where the elimination of absorbed water or ethanol took place. The second obvious thermal change appeared at 255 °C, where was lost another 12% weight due to the loss of hydrogen bonded water. With the increase of temperature, there was a sharp decrease in weight of ZnGC complex. The following event resulted in an endothermic peak at 360 °C with approximately 44% weight loss, which was related to the decomposition of ZnGC complex bonded with zinc and oxygen atom [37]. This thermogravimetric analysis of ZnGC, glucose and trisodium citrate dihydrate further confirmed the formation of ZnGC complex.

#### XPS

**Fig. 3:**
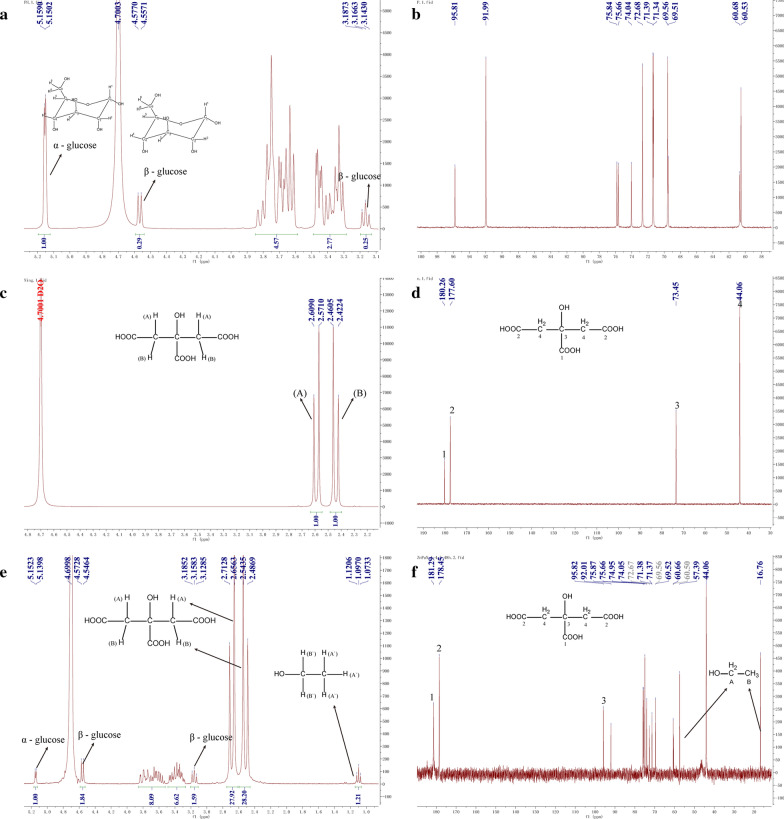
The ^1^H (**a**) and ^13^C (**b**) NMR spectra of glucose; the ^1^H (**c**) and ^13^C (**d**) NMR spectra of trisodium citrate dihydrate; the ^1^H (**e**) and ^13^C (**f**) NMR spectra of ZnGC

According to the results of XPS (shown in Fig. 2e and f), the composition elements of ZnGC complex were Zn, Na, O, and C. Zinc ion with citrate bound in the complex were in two binding modes. Glucose participates in the complex could form α-D-hexapyranose through hydrogen bond, and the trisodium citrate ligand forms the complex in acidic condition which is consistent with the results of NMR (Fig. 3) [33]. Therefore, the results confirmed the formation of ZnGC complex.

#### NMR analysis

The proton NMR spectra (^1^H and ^13^C) of glucose, trisodium citrate dihydrate and ZnGC complex were analyzed in D_2_O solvent (shown in Fig. 3). Glucose predominantly forms hexapyranose rings in aqueous solutions, which has two possible stereochemical isomers (due to presence of –OH group on C1 carbon). These two isomers were known as anomers and the anomerization of glucose in water occurs within a couple of hours. The population ratio of *α*- and *β*-D-glucose at equilibrium in water had been found to be 36:64 [38]. The data was in accordance with the rules in Fig. 3, where the ratio of *α*- and *β*-H was 1:2.04.

The NMR peaks of free trisodium citrate dihydrate ligand had been assigned as: H: δ(ppm) 2.59 (H(A), Fig. 3c, J = 12 Hz), 2.44 (H(B), J = 12 Hz); C: δ(ppm) 180.26(C1, Fig. 3d), 177.60 (C2), 73.45 (C3), 44.06 (C4). The ZnGC complex: δ(ppm) 2.655 (H(A), Fig. 3e, J = 18 Hz), 2.49 (H(B), J = 18 Hz); C: δ(ppm) 181.29(C1, Fig. 3f), 178.45 (C2), 74.97 (C3), 44.06 (C4). These assigned data confirmed that ZnGC complex had proton signals of trisodium citrate due to participation of carboxylate groups in the chelation process, which also changed the stereochemistry of free ligand to modify and apply it in terms of metal ions. No change or shift occurred in ^1^H and ^13^C NMR spectra of glucose, indicating that the glucose might cling to the complex through hydrogen bonding. Both FT-IR (Fig. 2a) and NMR results confirmed that trisodium citrate was coordinated with zinc(II) ions through oxygen of carboxylate groups while glucose was involved in the complex in the form of D-hexapyranose through hydrogen-bonding, which was also consistent with XPS results (Fig. 2e and f).

#### Fluorescence spectra

**Fig. 4:**
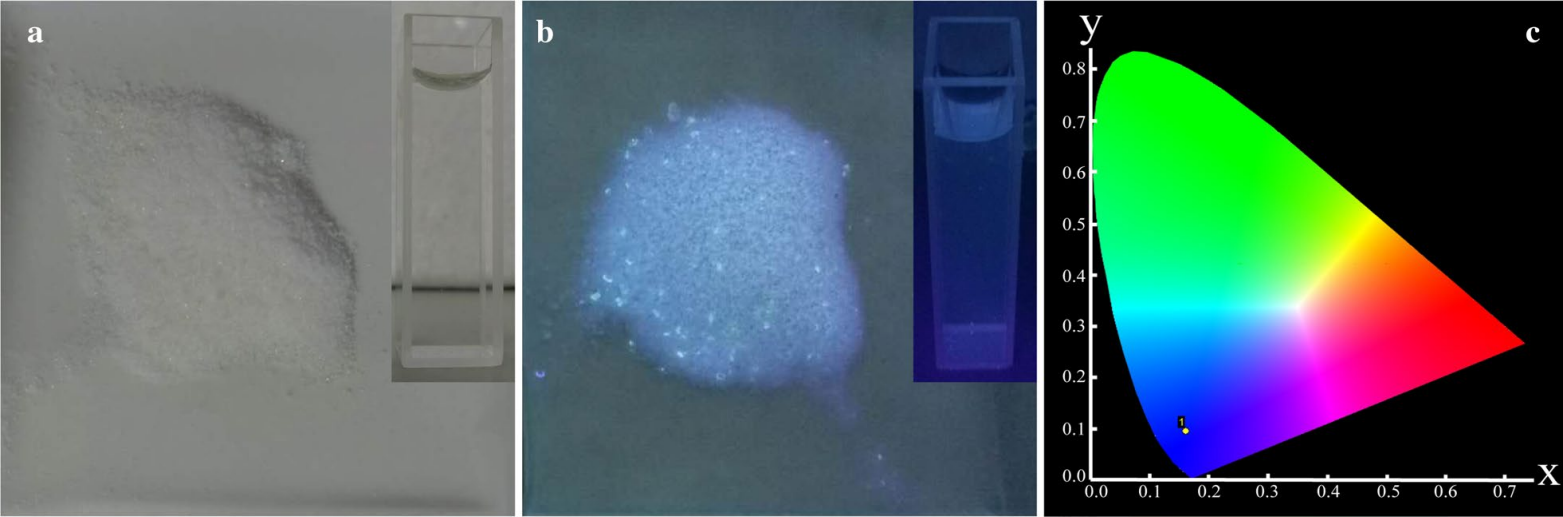
Images of ZnGC in sunlight (**a**); in 365 nm ultraviolet light (**b**); and the CIE chromaticity coordinates for ZnGC (**c**)

The fluorescence spectra of the ZnGC complex was recorded after heating the samples at 130 °C for 1 h in muffle furnace. The images and fluorescence phenomenon of pulverous and aqueous ZnGC complex in sunlight and in 365 nm ultraviolet light are shown in Fig. 4a and b, respectively. The emission spectrum of the ZnGC complex excited at 360 nm, showed an emission peak at 440 nm. The aqueous solution of the heated ZnGC complex was colourless under sunlight, however a weak blue fluorescence was observed under 360 nm ultraviolet light. The fluorescence behaviour of the novel complex varies under different temperature from 110 to 190 °C, and it was found that the strongest fluorescence was observed at 130 °C for 1 h.

The blue fluorescence at 360 nm was not observed in the case of a mixture of ZnCl_2_, glucose and trisodium citrate dehydrate under same condition or similar conditions, such as different pH, heating temperature and time etc. (data not shown). The generation of fluorescence property of the complex after heating, might be due to π-bond formation and there by resulting the energy transfer between ligand and Zn^2+^ ion. The new fluorescent band was considered to be related to the intermolecular charge transfer between π*–π and zinc ion. The behaviour of Zn^2+^ coordinated to the ligand was in general as that of emissive species leading to a CHEF effect (chelation enhancement of the fluorescence emission) [39]. This phenomenon also confirmed the formation of new ZnGC complex.

### Antioxidant activity

**Fig. 5:**
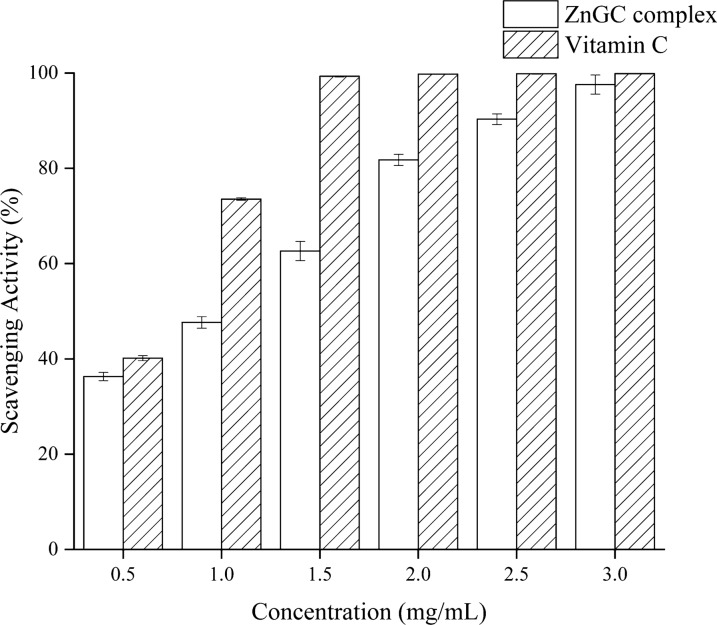
Hydroxyl radical scavenging activity (HRS%) of ZnGC at different concentrations

The hydroxyl radical scavenging activity (HRS%) was conducted to assess the antioxidant activity of ZnGC complex compared with vitamin C. There is no antioxidant activity of ZnCl_2_, thus the data not shown. As shown in Fig. 5, the antioxidant activity gradually increased with increasing the concentration of ZnGC complex and reached 99% at 3.0 mg/mL. The IC_50_ was 1.04 mg/mL. The DPPH (1,1-diphenyl-2-picrylhydrazyl) free radical scavenging activity and ferric ion reducing antioxidant power (FRAP) of ZnGC complex were also tested. However, no scavenging ability exhibited to organic free radicals and superoxide anions. The results showed that hydroxyl groups participated in the coordination while the complex formed [41].

### Antibacterial activity and MIC

Gram-positive and Gram-negative bacteria are responsible for infecting patients suffering from different diseases, such as trauma. These exhibit persistent adaptability and resistance to multi-drugs [40]. It is essential for the continuous improvement of antimicrobial materials that can effectively inhibit the growth of bacteria. Accordingly, we investigated the antibacterial activities of ZnGC complex against *S. aureus* (Gram-positive) and *E. coli* (Gram-negative) bacteria by agar plate well diffusion method. The ZnGC samples were placed in agar plate wells and the inhibition zones were analyzed in screening test. ZnGC complex showed excellent antibacterial activities against both targeted bacteria (shown in Table 1).

**Table 1:** Antibacterial inhibition zones of ZnGC against *S. aureus* and *E. coli*

ZnGC (mmol L^−1^) (zinc concentration)	Inhibition zones (mm)		ZnCl_2_ (mmol L^−1^) (zinc concentration)	Inhibition zones (mm)	
	*S. aureus*	*E. coli*		*S. aureus*	*E. coli*
184.0	27.8 ± 0.4	25.0 ± 0.4	184.0	19.0 ± 0.2	18.6 ± 0.3
92.0	23.5 ± 0.5	22.0 ± 0.5	92.0	16.0 ± 0.2	15.2 ± 0.1
46.0	17.9 ± 0.4	16.7 ± 0.5	46.0	11.5 ± 0.2	13.0 ± 0.2
23.0	13.1 ± 0.5	12.8 ± 0.4	23.0	8.9 ± 0.1	9.0 ± 0.2
11.5	9.4 ± 0.3	7.8 ± 0.3	11.5	–	–

The data are displayed as mean ± SD

The inhibition zones increased as the concentration of ZnGC increased. According to Wahid et al. [28] and Dutta et al. [41], the inhibitory effect of ZnGC complex might depend on Zn(II) which released and/or the reactive oxygen species (ROS) produced in the solutions. ZnGC complex could adhere with the negatively charged bacterial cell walls via electrostatic forces, damage cell walls and subsequently bind with proteins and nucleic acids of bacteria, which finally causes the cellular distortion and the loss of viability. ZnGC complex was formed by self-assembly through coordination bond between multi dentate organic ligand and central zinc ion. Similarly, another possible theory is that the production of reactive oxygen species (ROS) could mediate the membrane lipid oxidation of bacteria.

Therefore, the border of bacteriostatic ring of ZnGC was indistinct as compared to ZnCl_2_. The inhibition zone of ZnGC complex was bigger than ZnCl_2_ (having same molality of zinc ion), indicated that the antibacterial activity of ZnGC complex was higher than that of ZnCl_2_.

Minimum inhibitory concentration (MIC) is regarded as the minimum concentration of any material which inhibits the growth of the microorganism. Table 1 shows the bacterial activity of ZnCl_2_ and ZnGC complex. The MIC of ZnCl_2_ against *S. aureus* and *E. coli* were recorded as 13.5 and 13.5 mmol L^−1^ (zinc ion concentration) respectively. The MIC of ZnGC against *S. aureus* and *E. coli* were recorded as 10.0 and 10.0 mmol L^−1^ (zinc ion concentration) respectively. It indicated that ZnCl_2_ and ZnGC had similar effective against the two strains. And the results also showed that the antibacterial activity of ZnGC complex was higher than ZnCl_2_.

### Acute toxicity

Mice gavaged with ZnGC complex did not show any significant changes to their behavior as observed by respiratory distress, emaciation, posture, mortality, in behavioral, autonomic and toxic responses. All animals acted well without visible changes, and no animal mortality was observed during 14-days observation period under all the doses by oral administration. Body weight fluctuated in normal range and anatomical examination showed no signification difference between administration and control group (P > 0.05). The results indicated that ZnGC complex showed no obvious toxicity in this test, which indicated that it would be safe and reliable under the prescribed dose. (Ministry of Health, P.R. China, 2014). Therefore, ZnGC complex has the potential to be used as dietary supplements.

### In situ intestinal absorption study

**Fig. 6:**
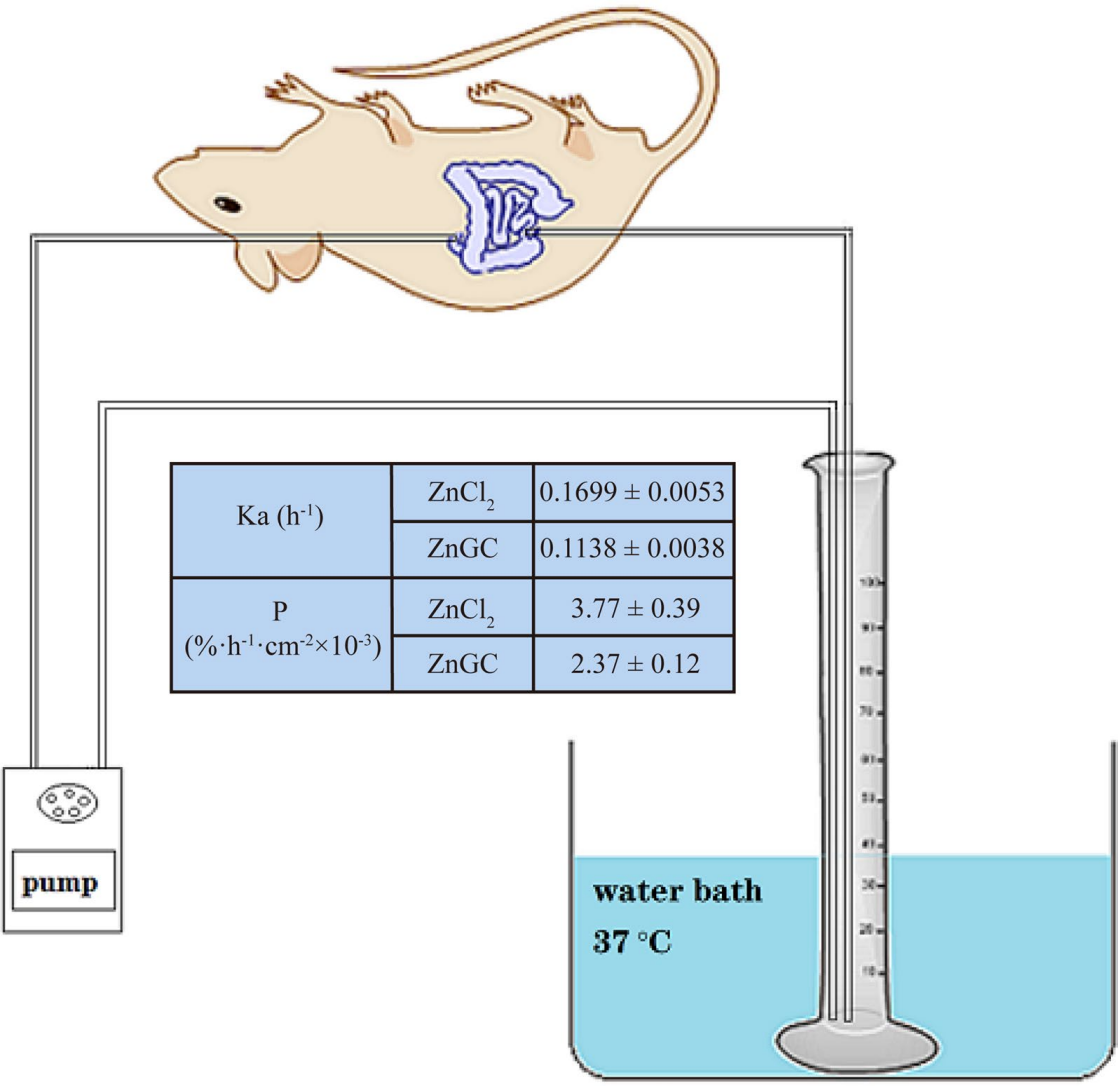
In situ intestinal absorption study: absorption rate constant Ka, and percentage P of ZnCl_2_ and ZnGC

The absorption rate constant Ka of ZnCl_2_ and ZnGC were 0.1699 and 0.1138 h^−1^ respectively. The absorption percentage P of ZnCl_2_ and ZnGC were 3.77% h^−1^ cm^−2^ × 10^–3^ and 2.37% h^−1^ cm^−2^ × 10^–3^ respectively (Fig. 6). The Ka and P of ZnCl_2_ were both higher than those of ZnGC, indicating that the absorption rate of ZnCl_2_ was faster. The results may be caused by free Zn^2+^ in ZnCl_2_ in solution which was easier to bind to transport protein. The absorption of ZnCl_2_ in the intestinal tract was dominated by active transport. It also suggested that ZnGC complexes adsorbed in molecular form leading to slow absorption rate and reduce gastrointestinal irritation. Although the absorption rate of ZnGC was lower, the value of Ka and P indicated that ZnGC still could be absorbed quickly, suggesting that ZnGC complex can be used as a potential zinc supplement for zinc deficiency, with lower gastrointestinal irritation and toxicity compare with ZnCl_2_.

## Conclusion

In this study, a novel ZnGC complex with anti-bacterial properties was synthesized and characterized. The zinc content of ZnGC complex was approximately 18.02%. FT-IR spectra showed obvious new peaks formed at 1394.52, 1604.72 and 3410.98 cm^−1^ in ZnGC complex, which significant changed from glucose and trisodium citrate dihydrate. Thermal analysis, XPS and fluorescence also showed the formation of novel ZnGC complex. The results of NMR confirmed that zinc ion was connected with trisodium citrate by coordinate bond, and glucose was linked through intermolecular hydrogen bonding to form ZnGC complex. Compared with ZnCl_2_, ZnGC complex had better bacteriostatic effect with lower toxicity. This novel zinc(II) complex presented similar antioxidant activity of hydroxyl radical as that of vitamin C at low concentration. In situ intestinal absorption study showed that ZnGC formed complexes with zinc adsorbed in molecular form and had low irritation to gastrointestinal tract and toxicity. Therefore, ZnGC complex has a potential to be used as a candidate for zinc supplements, application in clinic antibiosis material and biomedical fields.

## Data Availability

The datasets used and analyzed during the current study are available from the corresponding author on reasonable request. All methods were carried out in accordance with relevant guidelines and regulations. The study was carried out in compliance with the ARRIVE guidelines.

## Abbreviations

ZnGC: Zinc-glucose-citrate complex
SEM: Scanning electron microscopy
EDS: Energy dispersive spectrometer
NMR: Nuclear Magnetic Resonance
TEM: Transmission electron microscopy
FT-IR: Fourier transform infrared spectroscopy
XRD: X-ray diffraction
XPS: X-ray photoelectron spectroscopy
TGA: Thermal gravimetric analysis
HPLC: High-performance liquid chromatography
*S. aureus*: *Staphylococcus aureus*
*E. coli*: *Escherichia coli*
DAD: Diode array detector
MIC: Minimum inhibitory concentration
HRS: Hydroxyl radical scavenging activity
FRAP: Ferric ion reducing antioxidant power
DPPH: 1,1-Diphenyl-2-picrylhydrazyl
ROS: Reactive oxygen species
